# Velocity dependence of sliding friction on a crystalline surface

**DOI:** 10.3762/bjnano.8.218

**Published:** 2017-10-19

**Authors:** Christian Apostoli, Giovanni Giusti, Jacopo Ciccoianni, Gabriele Riva, Rosario Capozza, Rosalie Laure Woulaché, Andrea Vanossi, Emanuele Panizon, Nicola Manini

**Affiliations:** 1 Dipartimento di Fisica, Università degli Studi di Milano, Via Celoria 16, 20133 Milano, Italy; 2 Istituto Italiano di Tecnologia, via Morego 30, 16163 Genova, Italy; 3 Laboratoire de Mécanique, Département de Physique, Faculté des Sciences, Université de Yaoundé I. B.P. 812, Yaoundé, Cameroun; 4 CNR-IOM Democritos National Simulation Center, Via Bonomea 265, 34136 Trieste, Italy; 5 International School for Advanced Studies (SISSA), Via Bonomea 265, 34136 Trieste, Italy

**Keywords:** atomic-scale friction, contact, dissipation, friction, nanotribology, phonons, velocity dependence

## Abstract

We introduce and study a minimal 1D model for the simulation of dynamic friction and dissipation at the atomic scale. This model consists of a point mass (slider) that moves over and interacts weakly with a linear chain of particles interconnected by springs, representing a crystalline substrate. This interaction converts a part of the kinetic energy of the slider into phonon waves in the substrate. As a result, the slider experiences a friction force. As a function of the slider speed, we observe dissipation peaks at specific values of the slider speed, whose nature we understand by means of a Fourier analysis of the excited phonon modes. By relating the phonon phase velocities with the slider velocity, we obtain an equation whose solutions predict which phonons are being excited by the slider moving at a given speed.

## Introduction

Friction affects a wide variety of phenomena spanning broad ranges of length and time scales. Due to its practical and technological relevance, the study of friction was addressed even long before physics became a science. Fundamental steps in understanding its microscopic nature were achieved in the early 20th century [1–5]. Since the last decades of that century, major advancements in capturing the intimate relations between the atomistic dynamics and the dissipation mechanisms came as the result of the development of the atomic force microscope (AFM) and its friction force microscope (FFM) variant [6–8], as well as the extensive usage of atomistic molecular dynamics (MD) simulations and modeling made possible by the vastly increased computing power availability [9–16].

Despite this substantial progress, several fundamental mechanisms of friction and dissipation still need clarification. In particular, nanofriction (the study of friction at the atomic scale) lacks a theoretical model capable of accounting quantitatively for the dissipation of energy, i.e., the transformation of the kinetic energy of a macroscopic ordered motion into the internal energy of disordered thermal phonons. Both standard basic models of nanotribology, namely the Prandtl–Tomlinson model [1–2,13] and the Frenkel–Kontorova model [13,15–18], implement dissipation through a phenomenological viscous damping term acting on the physical degrees of freedom of the model. Moreover, such damping terms, beside affecting the dynamics, are characterized by a damping rate γ whose value is left to the arbitrary choice of the researcher. Finite-temperature is often simulated in the standard Langevin scheme, where to the viscous term one adds suitable Gaussian-distributed random forces, whose amplitude, via the fluctuation–dissipation theorem, is also affected by the value of γ [19].

A few approaches try to get rid of the arbitrariness of damping terms, describing dissipation explicitly. Microcanonical conservative simulations, for example, can describe the energy transfer into internal vibrational energy omitting all unphysical damping terms altogether [20–25]. The disadvantage of this approach is that, due to the finite and relatively small number of degrees of freedom available in a practical MD simulation cell, the dissipated energy accumulates in the phonons in the simulated sample, leading to a progressive overheating. Therefore this simulation procedure cannot address a steady sliding regime, but can at most identify the leading instabilities occurring and the most strongly coupled internal modes of the system. Another kind of approach involves using microcanonical equations to simulate a relatively broad cell including and surrounding the sliding contact region, adding dissipation through a suitable thermostat affecting only the atoms at the outer boundary of this region [26–35]. The main difficulty here is to provide a satisfactory implementation of the thermostat that not only provides a correct canonical dynamics in equilibrium conditions, but also manages to dispose of the extra excitation energy carried by the traveling phonon waves generated at the sliding contact, with minimal reflections at the boundary interface between conservative and thermostated particles. Such an implementation is in the process of being achieved [36–38], although with a rather intricate formalism, and should eventually become the technique of choice when one attempts to predict the sliding properties of a given physical interface for which a reliable force field is available. A third approach involves an uniformly distributed viscous damping term acting however only on the degrees of freedom perpendicular to the sliding direction [10,39–40]. While this method is quite effective in the context of fluid boundary lubrication, in the context of dry friction and crystalline interfaces nontrivial couplings of longitudinal and transverse modes [32,41–42] may lead to undesired γ-dependent effects.

In the present work we propose a different, minimalist approach, very much in the spirit of the Prandtl–Tomlinson and Frenkel–Kontorova (FK) models. We introduce and characterize a minimal slider–substrate model that captures the microscopic essentials of sliding friction and dissipation mechanisms, without the need of introducing ad hoc uncontrolled dissipative terms. This model consists only of a point-like localized cursor representing, e.g., a sharp AFM tip, interacting with an atomistic elastic substrate. As the slider skates on the elastically deformable crystal, phonons are generated and propagate away from the point of interaction. In this way, the slider mechanical energy is converted into crystal vibrational energy without any artificial viscous damping term affecting the slider itself. Of course, without any mechanism for dissipating this vibrational energy, the elastic substrate would eventually heat up as discussed above. For this reason we do include a weak viscous damping term, but this term acts on the elastic substrate only. We make sure that the effect of this term is negligible for the target of this research: the energy transfer from the slider to the substrate. This model virtually allows us to dispose of the arbitrary viscous damping, and to study the intrinsic dissipation properties of a sliding interface, as a function of several physical quantities, the most interesting of which is the sliding speed. Earlier work also investigated the speed dependence of the kinetic friction force in several models [1,25,43–50]. Under suitable conditions, specific velocities emerged as characterized by large kinetic friction due to suitable resonant conditions, in particular when the washboard frequency matches some phonon modes of the harmonic chain in the FK model [25,43]. In the present model too, characteristic resonances emerge at special speeds, but the resulting resonances differ significantly from those found for the FK model which represents an extended (infinite) solid–solid interface excited at a single wavelength, that of the sinusoidal potential. The present model focuses rather on the excitations induced by a localized point-like contact, inspired by an AFM tip, which has therefore the potentiality to generate excitations at all wavelengths.

This paper is organized as follows. Section “The Model” introduces our model. In Section “The kinetic friction force” we evaluate the kinetic friction as a function of the slider velocity. On top of a generally decreasing friction with sliding velocity, we identify peculiar “resonant” friction peaks at specific sliding velocities; in particular Subsection “Understanding: phonon phase velocities” draws a connection between the slider speed and the substrate phonon phase velocities, and proposes a relation that predicts the excited phonon modes when the slider advances at a given velocity, accounting for the observed dissipation peaks. In Section “Discussion and Conclusion” we discuss the obtained results, compare them to previous work, and sketch future extensions of the model. Appendix “The static friction force” reports and discusses the evaluation of the static friction threshold of this model.

## The Model

**Figure 1:**
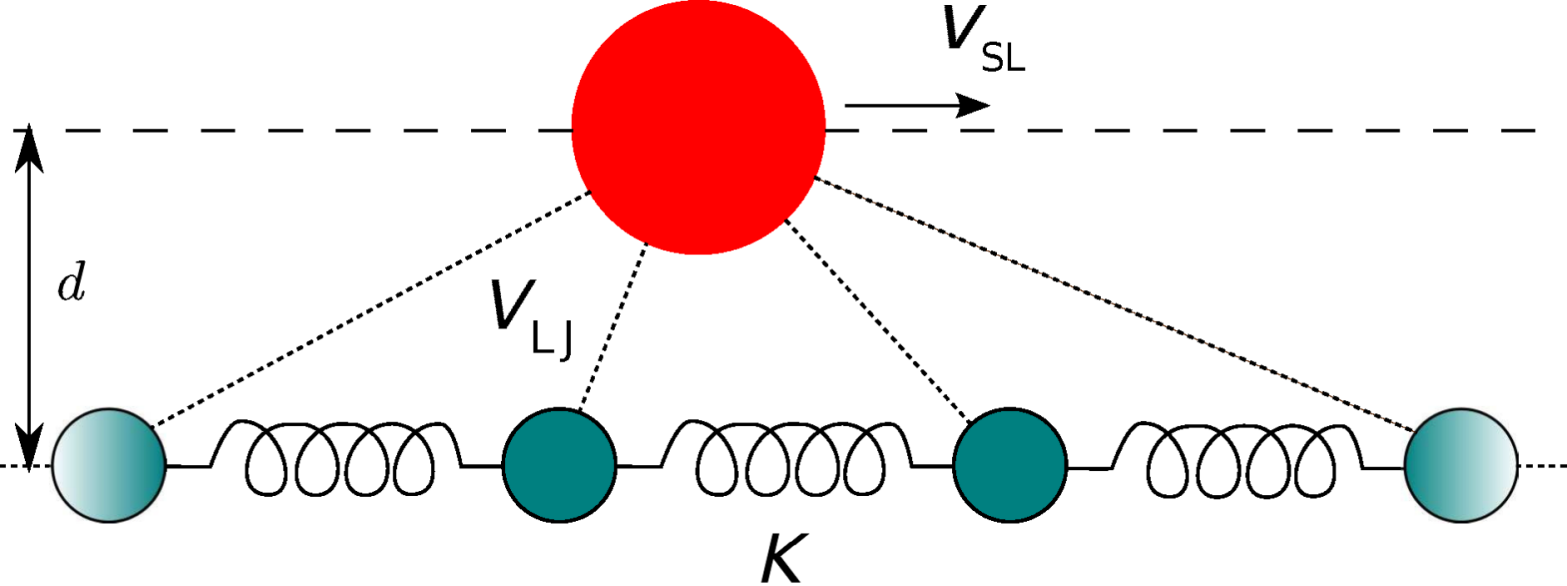
A sketch of the 1D slider-substrate model. The large sphere represents the slider, which moves along a fixed line (dashed) and interacts via Lennard–Jones forces with all atoms in the harmonic chain (smaller spheres).

We propose the model sketched, in its simplest form, in Figure 1. It consists of a slider plus a linear chain of *N* point-like atoms. The chain atoms, of mass *m* and positions *x_j_*, are connected by harmonic springs to their nearest neighbors. The total harmonic potential is

[1]
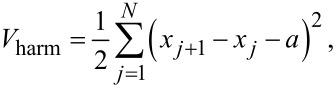


where *K* is the elastic constant of the springs and *a* is the equilibrium lattice spacing. The simulation is carried out inside a periodically repeated supercell of size *L* = *Na*, so that, e.g., in Equation 1 *x_N+_*_1_ ≡ *x*_1_.

The slider is also a pointlike particle, with horizontal position *x*_SL_ and mass *m*_SL_. We usually take *m*_SL_ *> m*, but this condition is by no means required. The slider follows a guide at a fixed distance *d* from the chain and interacts with each chain atom through a Lennard–Jones (LJ) term. Indicating with *R_j_* = [*d*^2^ + (*x*_SL_ − *x_j_*)^2^]^1^*^/^*^2^ the distance between the slider and the *j-*th particle, the total slider-chain interaction energy is

[2]
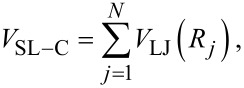


where *V*_LJ_ is the LJ potential

[3]
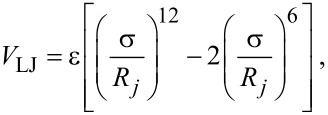


with equilibrium distance σ. The total potential energy *V*_harm_ + *V*_SL−C_ combined with the appropriate kinetic terms governs the model dynamics, which can be either classic or quantum. In Section “Discussion and Conclusion” we will discuss a few straightforward generalizations of this model, namely the removal of the constraint of a fixed slider–chain distance; a structured slider consisting of many atoms; the chain replaced by a 2D or, more realistically, 3D crystal.

A sinusoidal wave propagating in the chain follows the textbook dispersion relation [51] between its angular frequency ω and its 1D wave vector *k*:

[4]
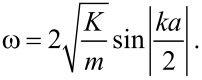


The corresponding long-wavelength speed of sound is

[5]
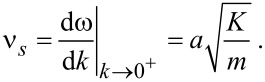


As discussed in the Introduction, the chain atoms are also affected by a weak viscous force, so that the phonon waves that propagate in the crystal get dampened and eventually fade while they move away from the point where they were generated, namely in the vicinity of the slider. The viscous force that acts on the *j*-th atom of the chain is

[6]
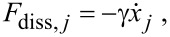


where γ is the damping coefficient. Thanks to this term, the oscillations generated by the slider are confined in a region smaller than the supercell, thus preventing the waves from coming back to the slider position.

In the present model all mechanical quantities can be expressed naturally in terms of the quantities *a*, *m*, and *K* characterizing the linear chain. Table 1 lists the natural units for the various mechanical quantities studied in the present work. For example, velocity is measured in units of the chain speed of sound *v_s_*: this means that, e.g., a slider speed |*v*_SL_| exceeding unity is a supersonic speed.

**Table 1:** Natural units for several mechanical quantities in the model.

Physical quantity	Natural units

length	*a*
mass	*m*
spring constant	*K*
force	*Ka*
energy	*Ka*^2^
time	(*m*/*K*)^1^*^/^*^2^
speed	*a*(*K*/*m*)^1^*^/^*^2^ ≡ *v_s_*
damping coefficient	(*mK*)^1^*^/^*^2^

**Figure 2:**
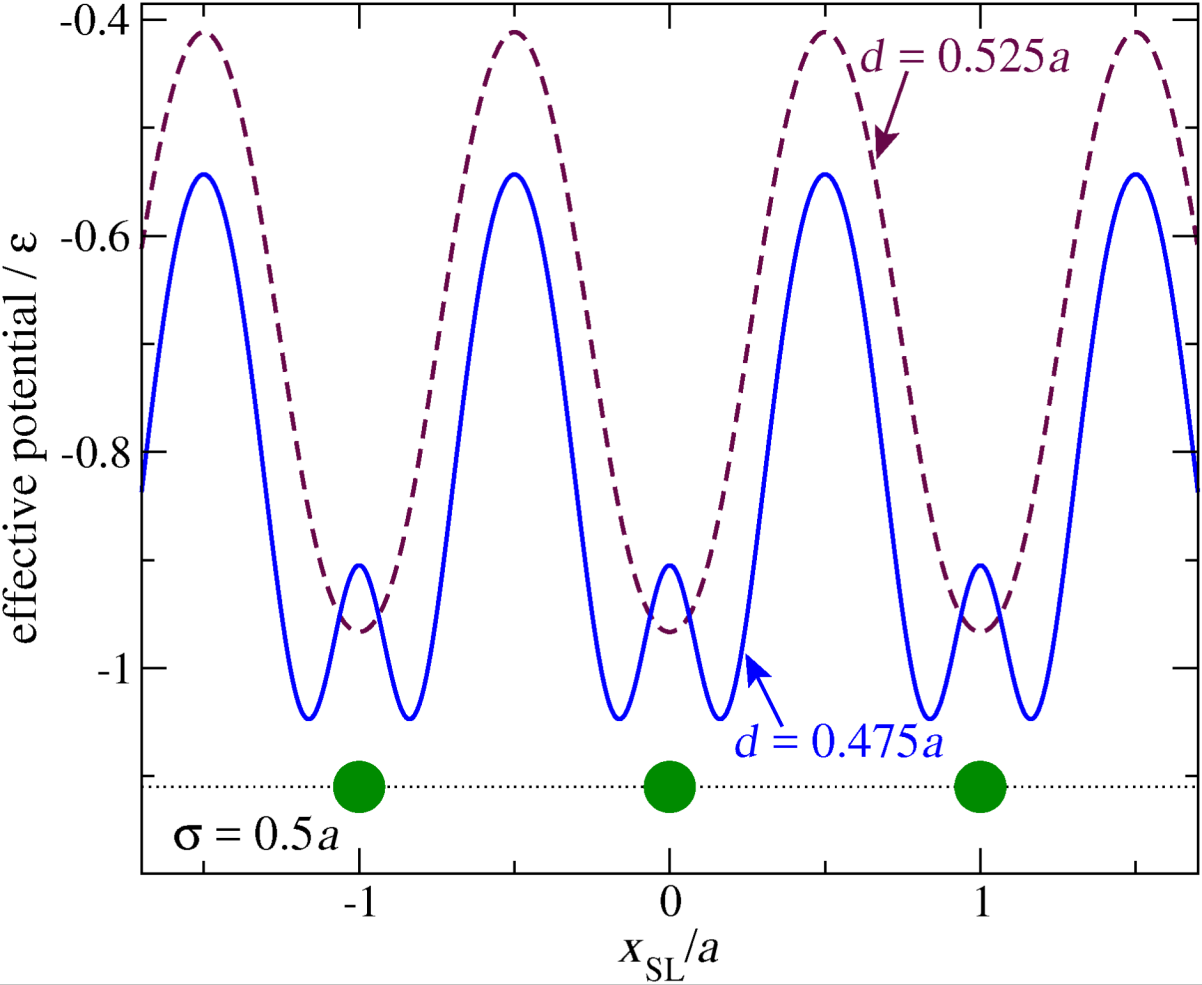
The potential-energy profile experienced by the slider as it moves along a hypothetical chain with atoms frozen at positions *x_j_* = *ja* (big circles), for a LJ parameter σ = 0.5*a*. Dashed curve: as obtained with *d* = 0.525*a >* σ; solid curve: with *d* = 0.475*a <* σ, the value adopted for the rest of this work.

In most simulations, unless otherwise specified, we adopt the following standard set of parameters: *L* = 500*a*, i.e., a chain formed by *N* = 500 atoms; a weak slider–chain coupling ε = 5 × 10^−4^ *Ka*^2^; slider mass *m*_SL_ = 10*m*; and chain damping γ = 0.1 (*mK*)^1^*^/^*^2^. We take a rather small LJ radius σ = 0.5*a*, and adopt an even closer approach distance between the slider and the chain *d* = 0.475*a*. The effect of this *d <* σ choice is to generate a periodic double-minimum effective potential experienced by the advancing slider in the limit where one could neglect the displacements of chain atoms, see the solid line of Figure 2. In contrast, a larger *d >* σ would rather produce a potential of the type depicted by the dashed line of Figure 2: for simplicity, we defer the investigation of the *d >* σ scenario to future work.

## The Kinetic Friction Force

### Simulation protocols

**Figure 3:**
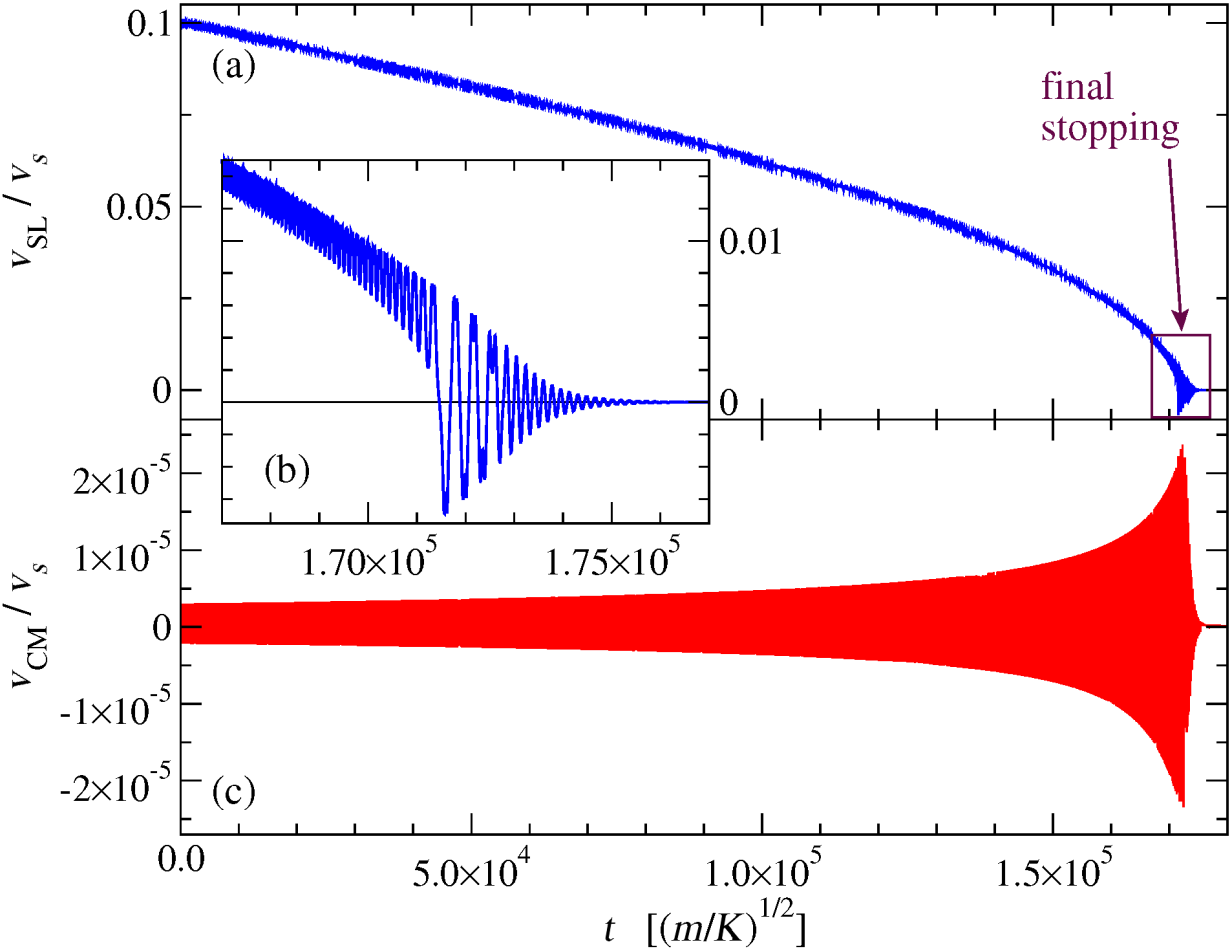
The time dependence (a) and (b) of the slider velocity *v*_SL_ and (c) of the chain center of mass *v*_CM_ for the second simulation (freely-slowing mode) of protocol A. The simulation is carried out until the slider finally stops and dissipates its residual energy in oscillations expanded in the inset (b), around a minimum of the effective potential of Figure 2, with chain phonon waves carrying this residual energy away, until the eventual complete arrest of the system. Note the increasing negative mean slope of the *v*_SL_ curve, indicating an increasing mean friction force *F_d_* as *v*_SL_ decreases.

To evaluate a meaningful estimate of the kinetic, or dynamic, friction force experienced by the advancing slider, we develop two alternative protocols [52–53]. In protocol A we first execute a “run-in” calculation at constant speed *v*_SL_, that allows the initial shock waves induced by the sudden apparition of the slider near the chain to die out. Starting from the dynamical condition reached at the end of the first simulation, we then run a second simulation in which the slider is allowed to change its velocity as a consequence of the interaction with the chain and its own inertia like, e.g., in “ballistic” experiments where clusters deposited at surfaces are kicked around until they come to rest dissipating their kinetic energy into the substrate [49,54–56]. Figure 3 displays an example of the time dependence obtained in such second simulation. From the slider mean slowing rate during this second simulation, one can extract the kinetic friction force with the following method: time is divided into regular intervals, e.g., of 5000 time units, a very long time compared to the period of the fluctuations of *v*_SL_; then a linear regression is performed over each interval; the (negative) slope of the straight line fitting *v*_SL_ as a function of time represents the slider average acceleration. The average dynamic friction force *F_d_* experienced by the slider is then obtained by multiplying this acceleration by (−*m*_SL_). Associating this value of *F_d_* to the mean value of *v*_SL_ in the same time interval, one obtains the dependence *F_d_*(*v*_SL_). The faster and faster slider slowing rate displayed in Figure 3a indicates an increasing dynamic friction *F_d_*(*v*_SL_) as its speed *v*_SL_ decreases. As *v*_SL_ decreases and friction increases, the chain center-of-mass velocity *v*_CM_ oscillates more and more, a useful indicator of a larger and larger transfer of momentum from the slider to the chain. Of course the observed small-amplitude oscillations of *v*_CM_ are almost entirely the result of the oscillations induced in the few chain atoms directly interacting with the slider, with the vast majority of far-away atoms remaining essentially stationary. These center-of-mass oscillations are therefore an artifact of the finite size of the chain, and would vanish entirely in the macroscopic-size limit.

Protocol B is as follows: we execute a single calculation keeping *v*_SL_ constant, as if the slider was an AFM tip attached to an infinitely rigid AFM cantilever, wait until a steady-sliding regime is established, and discard the initial part affected by transients. For the remaining part of the simulation, we record the total force experienced by the slider as a function of time. This force has fluctuations as a result of collisions against the consecutive atoms of the chain. The period of these fluctuations is *a*/*v*_SL_. We average this force over an integer number of these periods, and interpret the result as the average friction force *F_d_* corresponding to the velocity *v*_SL_. We start over a new simulation for every value of *v*_SL_ of interest.

While protocol A evaluates rigorously the friction force, allowing for all kinds of acceleration–deceleration effects related to individual slider–atom collisions, protocol B would coincide with protocol A only in the limit where the energy transferred in a single atomic collision is much smaller than the kinetic energy accumulated in the slider. The LJ parameter ε provides a conservative estimate of the energy transferred in the collision: the condition for a rigorous applicability of protocol B is

[7]
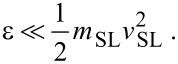


This means that protocol B should yield results identical to protocol A for *v*_SL_ ≥ (2 ε/*m*_SL_)^1/2^ 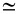 10^−2^ *v_s_* for the standard simulation parameters introduced above. We have verified numerically that this is indeed the case. We have also verified numerically that in the considered weak-coupling regime, *F_d_* 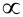 ε^2^. This is in accord with the observation that the dissipated power *F_d_v*_SL_ must equal the average work per unit time done by the slider on the chain. The latter equals the product of the force exerted by the slider on each chain atom times that atom’s velocity *v_j_*. The force is of course proportional to the coupling ε and, to first order in ε/(*Ka*^2^), also *v_j_* is proportional to ε, whence the *F_d_* 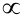 ε^2^ dependence at weak coupling.

Incidentally note that, in both protocols, the slider transfers not only energy but also momentum to the chain. Indeed the friction force *F_d_* equals precisely the transfer of momentum per unit time from the slider to the chain. *F_d_* is negative (leftward) when considered acting on the slider; due to the Newton’s third law, the chain experiences a positive (rightward) force of precisely the same intensity. As a combined result of *F_d_* and the damping force provided by the dissipative terms of Equation 6, in the steady regime the slider acquires a small but nonzero mean center-of-mass velocity *v*_CM_. As a result, the slider speed relative to the chain is decreased relative to the imposed *v*_SL,abs_, precisely by 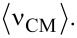 In the following, we denote by *v*_SL,abs_ the slider velocity in the lab frame, and by *v*_SL_ the slider velocity in the frame of the chain center of mass:

[8]
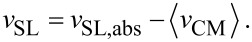


In practice, we obtain *v*_CM_ /*v*_SL,abs_ 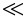 1 for not-too-small *v*_SL,abs_, so that this correction is irrelevant, and *v*_SL_ 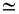 *v*_SL,abs_. However at low speed this correction can become significant.

### Control of the dissipation terms

**Figure 4:**
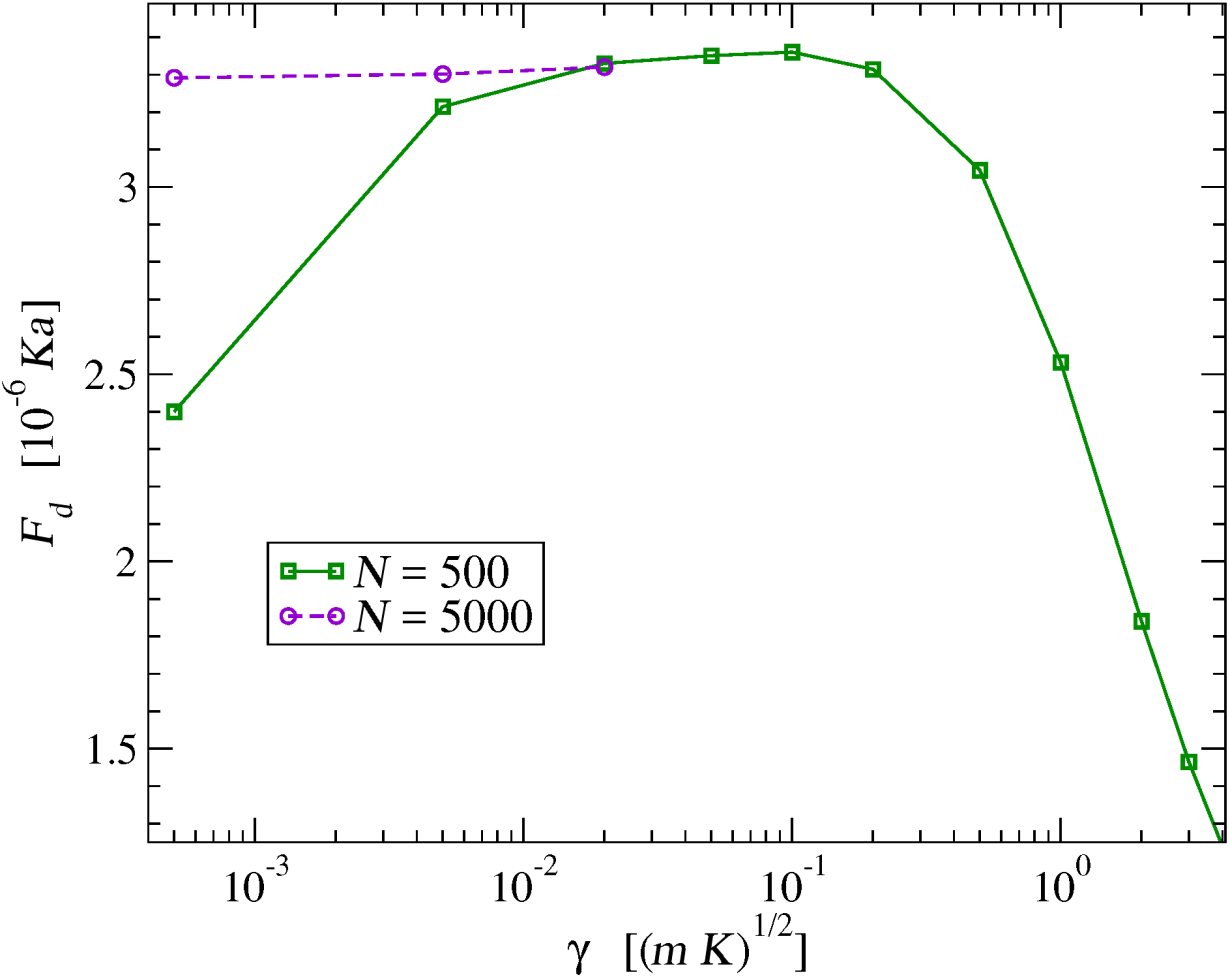
The dynamical friction force as a function of the damping coefficient γ that fixes the (unphysical) dissipation rate of the chain atoms. This is evaluated for *v*_SL_ = 0.1 *v_s_* with protocol A (but protocol B provides identical data). Squares: simulations executed in a standard supercell of size *L* = 500*a*, with 500 atoms. Circles: similar simulations executed in a 10× longer supercell with 5000 atoms, where the waves emitted by the slider have a much longer space to decay fully before coming back to the contact point, even for smaller γ.

**Figure 5:**
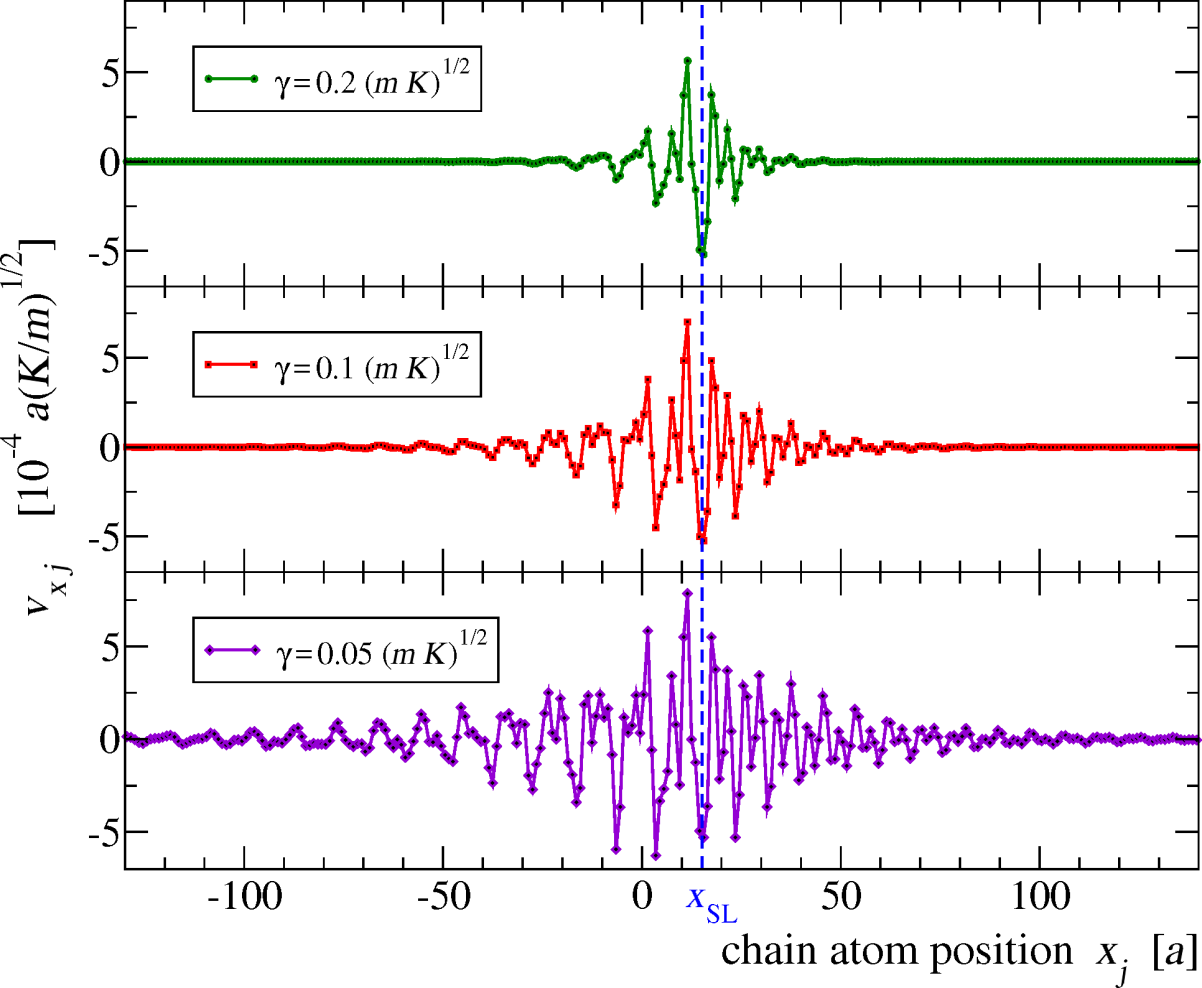
Three snapshots of the instantaneous velocities of a few chain atoms as a function of their position while interacting with the slider, whose instantaneous position *x*_SL_ is marked by a vertical dashed line. The three calculations are executed with the same *v*_SL_ = 0.1 *v_s_*, but with three different values of γ, which produce visibly different damping of the same excited phonon waves away from the interaction point.

First off, we need to identify a range for the dissipation parameter γ where the dissipative terms of Equation 6 become irrelevant to the frictional dynamics. Figure 4 displays the dependence of the sliding friction measured for *v*_SL_ = 0.1 *v_s_*, obtained repeating the simulations with different values of the damping parameter γ. This figure shows that for large and increasing γ, friction tends to decrease. This is expected, since in the unphysical strongly overdamped regime, it is harder and harder to displace the chain atoms away from their equilibrium positions, with the result that their effect on the slider resembles more and more closely that of a conservative effective potential, namely the one of Figure 2, which of course allows for no energy transfer, and no dissipation. As damping is decreased to the physically relevant underdamped region γ *<* (*mK*)^1^*^/^*^2^, friction stabilizes to a physically significant γ-independent value. However, when γ is further decreased below approximately 10^−2^ (*mK*)^1^*^/^*^2^, the waves produced at the slider–chain contact point become less and less damped, see Figure 5. When the γ value becomes too small, the phonon waves propagate all the way across the periodically repeated cell, eventually returning to the slider–chain contact point. As a result, the chain slides over an effectively “hot” substrate, generating an unphysical friction reduction related to thermolubricity [45–46,57–62]. As shown by the circles in Figure 4, a much longer chain provides enough space for the phonon waves to decay, thus allowing the phonon amplitude to be radically reduced before returning to slider-chain contact point, even with small γ. In the following we adopt γ = 0.1(*mK*)^1^*^/^*^2^ as the standard value of the damping rate, unless otherwise indicated.

### Sliding-speed dependence of friction

**Figure 6:**
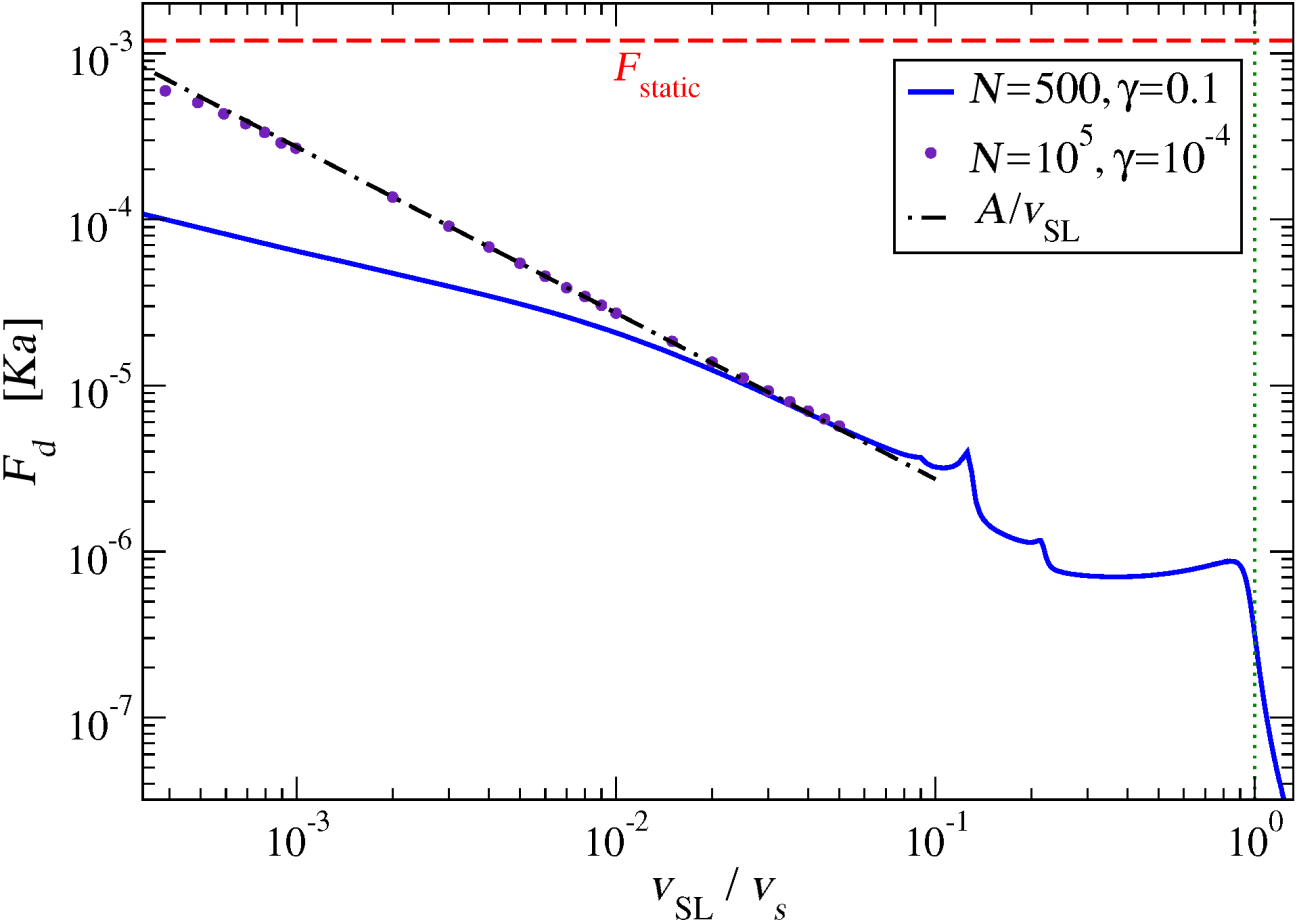
Dynamic friction force as a function of the slider speed *v*_SL_. For comparison, the horizontal dashed line marks the static-friction threshold *F*_static_ obtained in Appendix “The static friction force”, Equation 9. The vertical dotted line marks the speed of sound *v_s_*. The data are obtained by means of protocol B; we verified that perfectly superposable curves can be obtained by means of protocol A as long as it can be effectively applied, i.e., as long as the friction force *F_d_* *<* 10^−5^ *Ka*.

Figure 6 shows the speed dependence of the kinetic friction *F_d_*. The general trend is of a decreasing friction with speed. In particular, *F_d_* drops dramatically to 0 when *v*_SL_ exceeds the speed of sound of the chain. In the region below the speed of sound (vertical dotted line) *F_d_* stands on a relatively stable plateau. Several friction peaks then emerge in the region roughly between 10% and 20% *v_s_*. When *v*_SL_ is further decreased, friction starts to rise approximately as 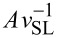 (where *A* is a constant), although, with the standard parameters of Section “The Model”, a deviation is observed quite early, for *v*_SL_ 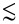 0.03 *v_s_*. This deviation is not particularly related to reaching the speed region where protocol B, introduced in Section “Simulation protocols” above, becomes unreliable. We have verified that this deviation is instead an artifact of the damping in the chain. By repeating the simulations with a longer chain (*L* = 10^5^*a*) and smaller damping γ = 10^−4^(*mK*)^1^*^/^*^2^, we find that the *F_d_* = 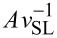 regime persists down to *v*_SL_ 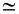 10^−3^ *v_s_*. With the adopted value for the coupling ε, protocol A cannot determine friction reliably as a function of *v*_SL_ in this region of speeds, because *v*_SL_ itself oscillates widely in time as the slider nears stopping, see Figure 3b. However we reduced the coupling ε by one order of magnitude to ε = 5 × 10^−5^ *Ka*^2^, and verified that the points of the *N* = 10^5^, γ = 10^−4^ (*mK*)^1^*^/^*^2^ curve are perfectly reproducible, apart from a factor 10^−2^ smaller friction, thus confirming the *F_d_* = 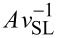 low-speed scaling.

As shown in Figure 3, the simulations of this model do provide concrete realizations of the dynamic to static transition. Unfortunately, due to the wide oscillations displayed in Figure 3b, the transition from dynamic to static friction is just impossible to characterize in terms of a simple function *F_d_*(*v*_SL_). We evaluate the static friction for this model as detailed in Appendix “The static friction force”. The comparison of *F_d_* and *F*_static_ in Figure 6 confirms that of course this model is consistent with the well-known result that *F_d_* *< F*_static_.

We should now investigate the reason for the friction peaks at certain special subsonic velocities. For this purpose, we must identify what phonon modes are most strongly excited at each particular slider speed.

### Phonon excitations

The friction force *F_d_* of Figure 6 dissipates the slider kinetic energy by transforming it into phonon waves that travel away from the contact point, as illustrated in Figure 5. We analyze these phonon excitations for several values of *v*_SL_. We must first detail the protocol of this analysis.

**Figure 7:**
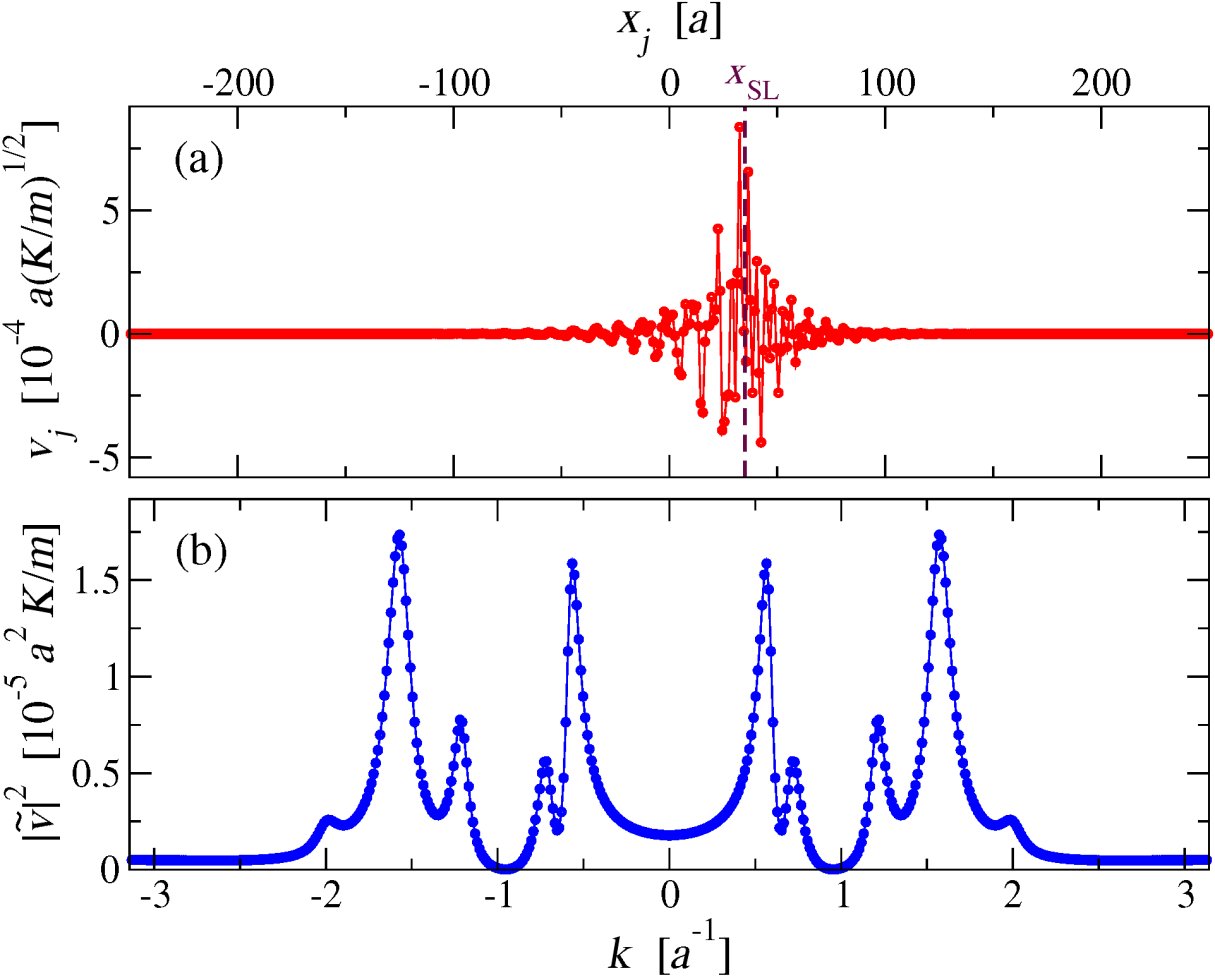
(a): A snapshot of the velocities of the chain particles while the slider, instantly at the vertical dashed-line position, advances at *v*_SL_ = 0.1 *v_s_*. (b): The square modulus of the spatial Fourier transform 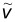
(*k*, *t*) of the velocities, as defined in Equation 10.

We run a simulation with fixed slider speed *v*_SL,abs_. At a given time *t* after the end of the initial transient, we take a snapshot of the individual velocities *v_j_* of the chain atoms, as in the example of Figure 7a. We then execute a spatial Fourier transform of these instantaneous velocities:

[10]
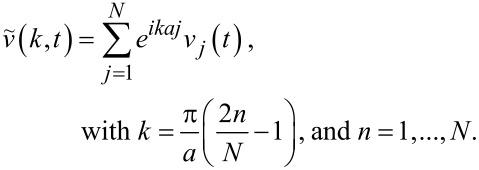


Here *i* is the imaginary unit and *k* spans the first Brillouin zone (BZ) (−π/*a*, π/*a*]. Figure 7b displays 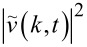 for the velocity pattern of Figure 7a. Observe first that the real values of *v_j_* guarantee that 
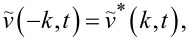
 and therefore that 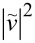 is an even function of *k*, as is evident in Figure 7b. Due to this symmetry in all following figures reporting Fourier transforms we will display the positive half [0, π/*a*] of the first BZ only.

The peaks of the Fourier transform highlight the phonons most excited by the interaction with the slider at that given instant in time. For a given *v*_SL_, during the simulation the velocity pattern *v_j_*(*t*), and therefore its Fourier transform, evolve in time. We take advantage of the fact that the slider encounters the chain particles at a regular interval *T* = *a*/*v*_SL_: in the steady state *v_j_*(*t*) is a periodic function of time but for a lattice translation, *v_j+1_*(*t* + *T*) = *v_j_*(*t*). As a global translation (a shift in *j*) only affects the phase of 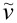
(*k*, *t*), but not its amplitude, 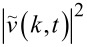 is exactly periodic in time:

[11]
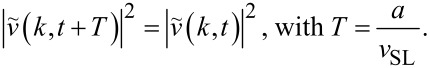


**Figure 8:**
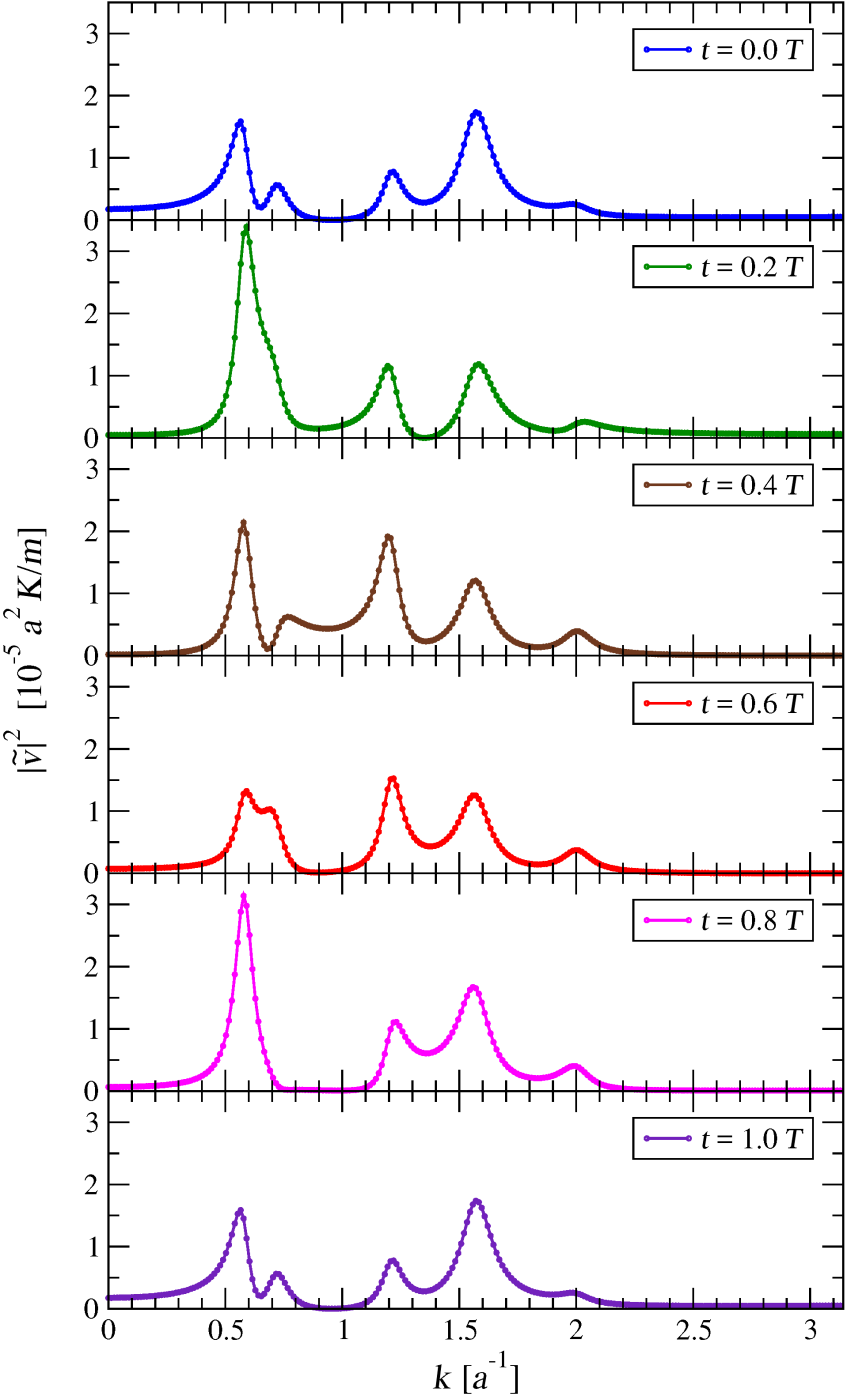
The square modulus of the Fourier transforms of the velocities *v_j_*(*t*), at six subsequent times. Here the slider velocity is *v*_SL_ = 0.1 *v_s_*. Comparing the plots of 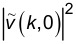 and 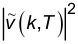, one sees that 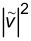 repeats itself identically after one entire period *T* = *a*/*v*_SL_ = 10 (*m*/*K*)^1^*^/^*^2^.

Figure 8 displays an example of this periodic time dependence of 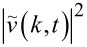.

To obtain a time-independent description of the typical phonon-excitation spectrum at a given *v*_SL_, we average the square modulus of 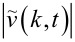 over a period *T*. We select *M* = 50 instants of time, equally spaced within the period, and compute 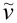 at each of them. Then we calculate the time-averaged Fourier transform as follows:

[12]
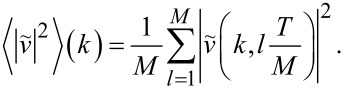


We verified that a finer sampling of the period (larger *M*) shows no significant difference in this average spectral intensity.

**Figure 9:**
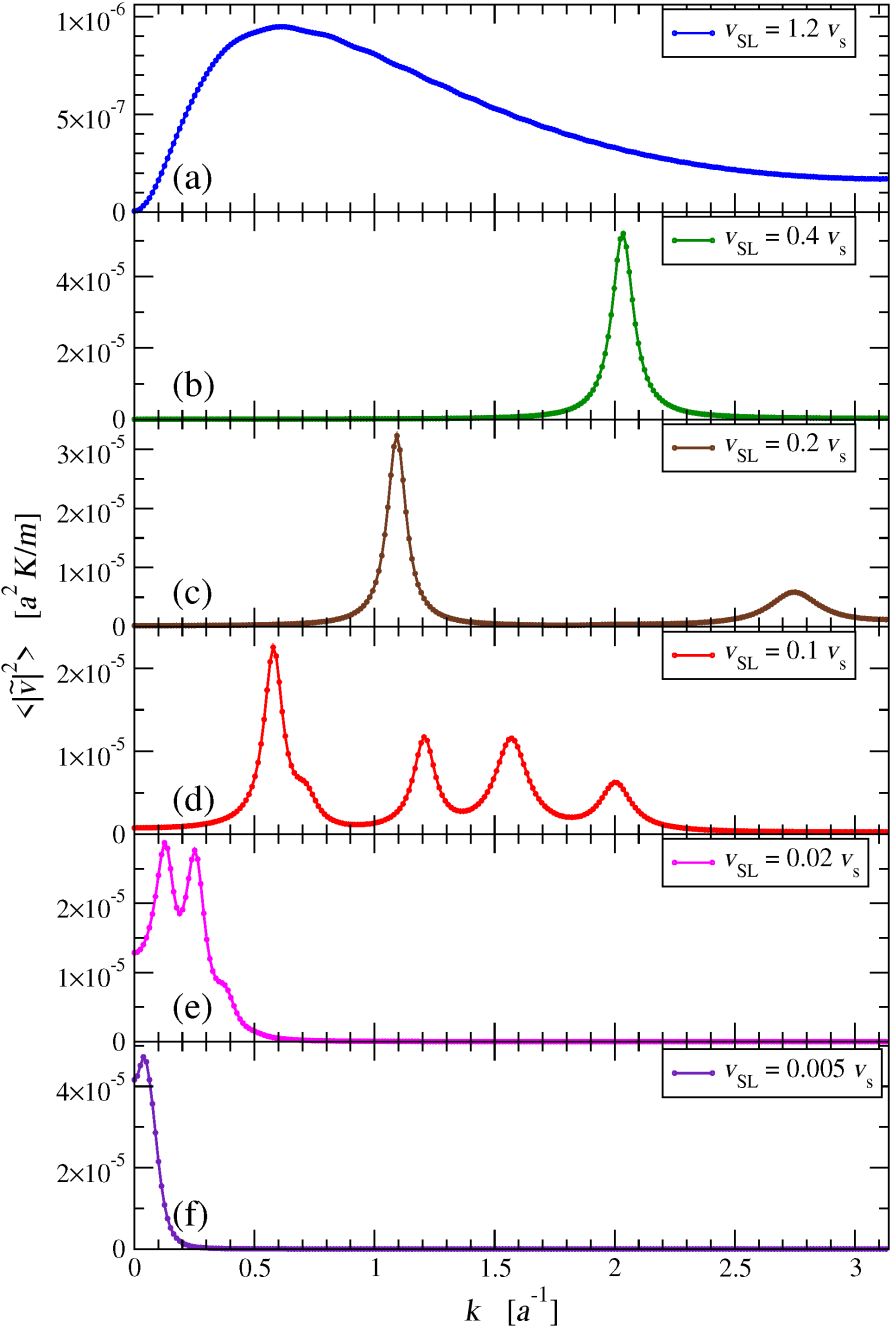
The time-averaged square modulus of the Fourier transform of the chain velocities, for a few characteristic values of *v*_SL_: (a) supersonic speed; (b) single-peak subsonic region; (c) appearance of the second pair of peaks; (d) multi peak region near *v*_SL_ 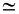 0.1 *v_s_*; (e) small-speed collapse of the dissipation peaks toward *k* = 0; (f) full collapse to one peak at very small *v*_SL_ 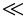 *v_s_*.

Figure 9 illustrates the resulting time-averaged power spectra 
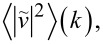
 for a few sample velocities *v*_SL_. Figure 9a shows that for supersonic *v*_SL_ the power spectrum is quite flat, with no sharp peak: most phonon modes are excited with comparable, rather weak, intensities; only those closest to *k* = 0 are left substantially unexcited. When *v*_SL_ is less than the speed of sound *v_s_*, but greater than 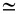
0.217 *v_s_*, as in Figure 9b, the spectrum is dominated by a single peak. As *v*_SL_ is lowered below 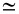
0.217 *v_s_*, as in Figure 9c,d, multiple peaks appear, and, as *v*_SL_ decreases, their number increases progressively. As *v*_SL_ further decreases, Figure 9e, the positions of all peaks approach *k* = 0, where they gradually merge into a single peak, Figure 9f. Note that the spectral resolution is limited both by the finite chain length, with δ*k* = 2π/(*Na*), see Equation 10, and by the phonon localization induced by the damping term of Equation 6.

**Figure 10:**
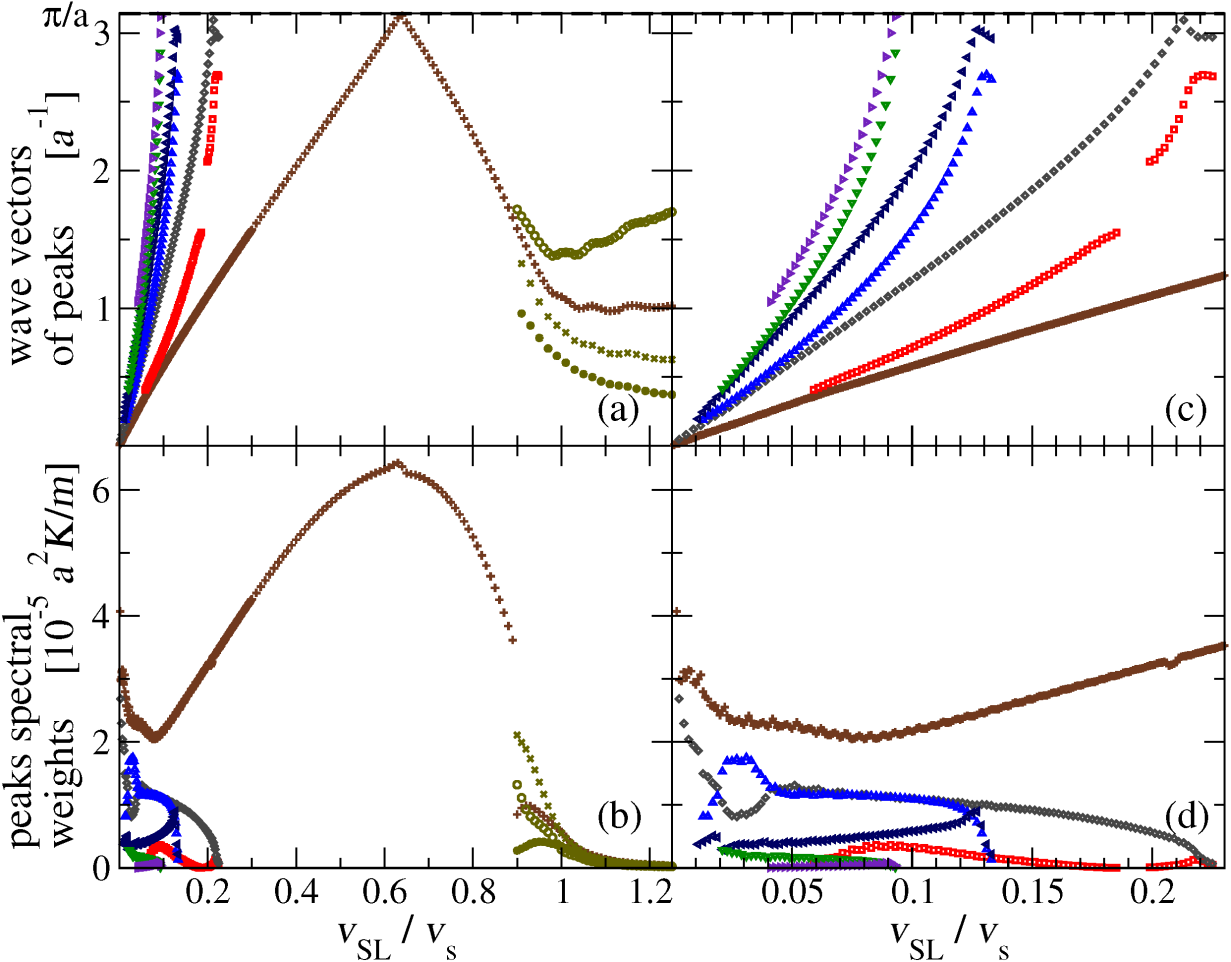
The positions (a) and heights (b) of the peaks of 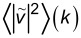 as functions of *v*_SL_. Panels (c) and (d): detail of the *v*_SL_ ≤ 0.23 *v_s_* low-speed region for the same quantities.

Figure 10 tracks the positions and intensities of the observed peaks of 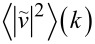 as functions of *v*_SL_. These data are obtained by fitting the functions 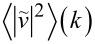 with sums of up to 7 Lorentzian curves. Figure 10 reports the centers and heights of the fitted Lorentzian terms.

Even in the supersonic region *v*_SL_ *> v_s_*, where the phonon power spectrum is smooth and flat and lacks sharp peaks as in Figure 9a, we fit the spectrum with the sum of four Lorentzian curves. These four Lorentzian profiles do their best to interpolate that continuum spectrum. Not surprisingly, the Lorentzian centers and intensities, displayed at the right side of Figure 10, follow a rather erratic variation with *v*_SL_, confirming that in the spectrum for supersonic sliding no sharp peak is actually present.

As *v*_SL_ decreases below approximately 90% of the speed of sound, initially a single prominent peak appears near the BZ boundary (Figure 9b), tracked in wave vector and intensity by the crosses in the central region of Figure 10a,b. As *v*_SL_ decreases below 0.217 *v_s_*, more peaks appear near the BZ boundary too, as in the examples of Figure 9c,d, and tracked explicitly in Figure 10c,d. As *v*_SL_ is further decreased all peaks move down toward *k* = 0 and eventually merge for *v*_SL_ 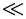 *v_s_*, due to the finite *k*-resolution (Figure 9e,f). Figure 10c clarifies a nontrivial feature of this velocity-dependent phonon spectrum: except for the single peak emerging at *v*_SL_ 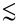 *v_s_*, all other new peaks appear in pairs.

**Figure 11:**
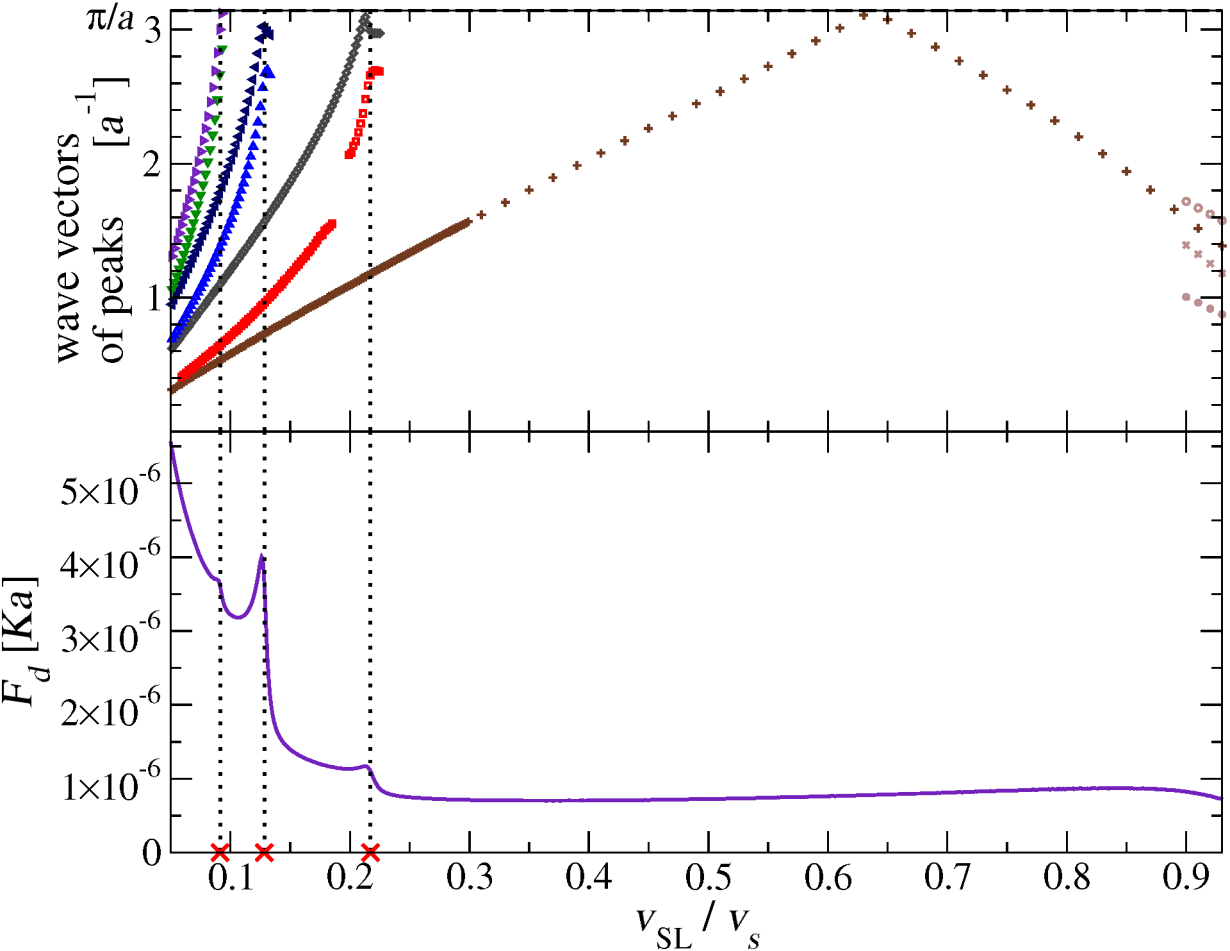
A direct comparison of the peak wave vectors of 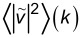, with the dynamic friction *F_d_* as a function of *v*_SL_ over the interesting speed range of Figure 6. The dotted vertical lines highlight the relation of the peaks of *F_d_* with the appearance of new pairs of excited phonons near the BZ boundary.

Figure 11 highlights the relation between the appearance of new peaks in the chain phonon spectrum of excitations at certain speeds with jumps in the dynamic friction *F_d_* as a function of *v*_SL_. The important conclusion of this correlation is the following: dissipation increases suddenly for those values of *v*_SL_ where new phonon modes start to get excited.

### Understanding: phonon phase velocities

Given the simplicity of this 1D model and the special weak-coupling regime investigated, we should be able to understand why certain phonons get excited at certain special speeds. We formulate the hypothesis that phonon modes get excited when their phase velocities ω/|*k*| match the slider speed *v*_SL_. We start by searching all values of *k* such that the phonon phase velocity matches a given speed *v*_SL_:

[13]
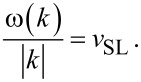


We substitute the simple dispersion relation ω(*k*) of Equation 4, and reformulate Equation 13 as:

[14]
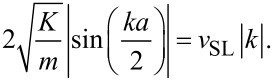


This equation is put in dimensionless form by introducing 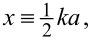 and the parameter 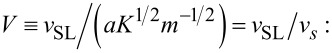


[15]
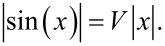


For a given *V* (or equivalently a given *v*_SL_), the solutions *x* (or equivalently *k*) of Equation 15 (or equivalently Equation 14) provide the special wave vectors such that the phonon phase velocity matches the given speed *v*_SL_.

**Figure 12:**
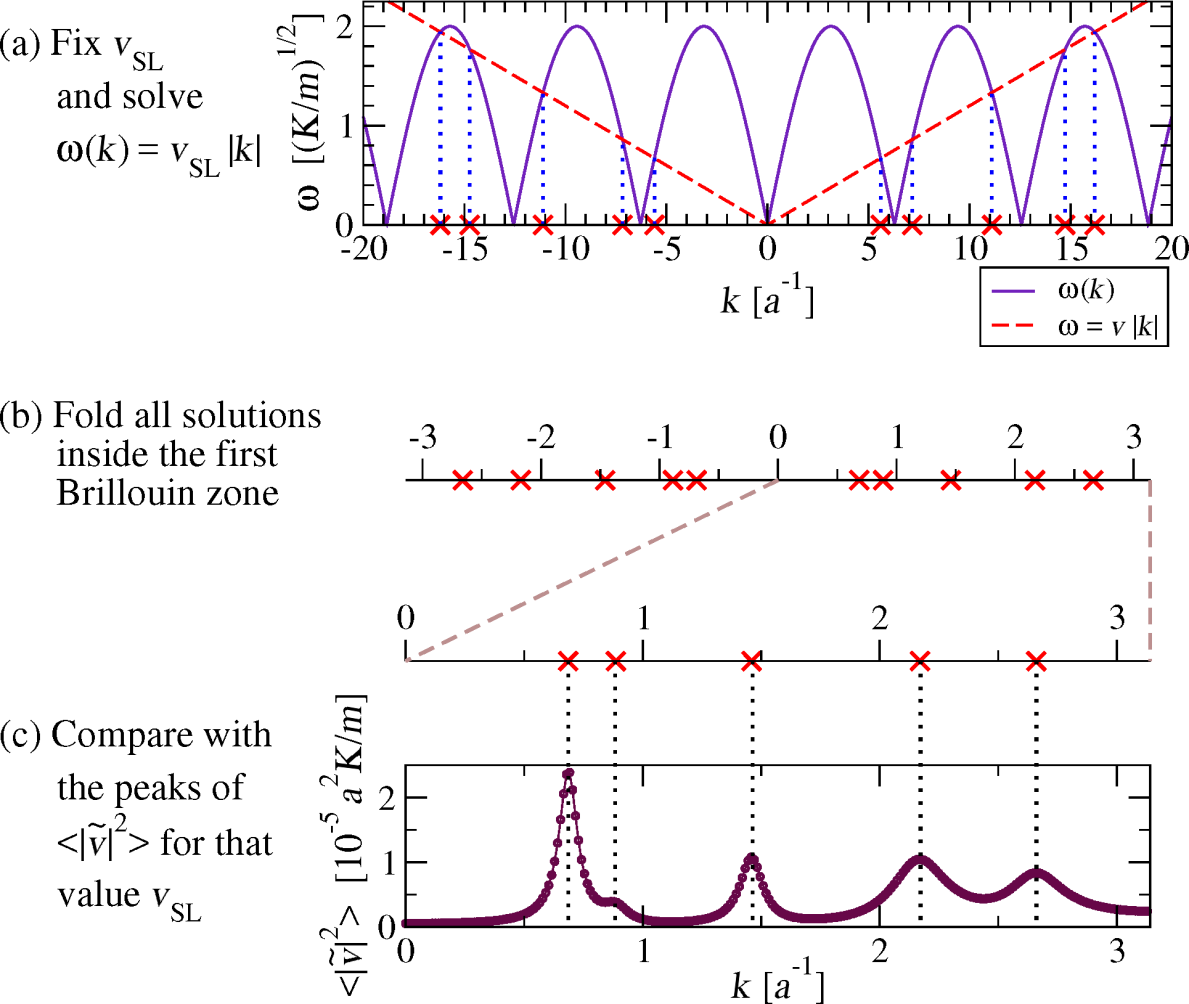
A scheme of the steps performed to identify the phonons whose phase velocity equals *v*_SL_, and the comparison of the obtained wave vectors with the observed excited phonons peaks for the same slider velocity *v*_SL_. In this example *v*_SL_ = 0.12 *v_s_*.

Equation 15 can of course be solved numerically for any *V*. An example of graphic solution of Equation 14 is displayed in Figure 12a. The resulting wave vectors are scattered over several BZs. We then bring all the *k*-point solutions inside the first BZ, Figure 12b. Finally, we proceed to compare these solutions to the positions of the peaks of the power spectrum 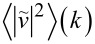 obtained by MD at that specific slider speed, Figure 12c. In the example of Figure 12, this procedure is followed for *v*_SL_ = 0.12 *v_s_*, showing that the solutions of Equation 14 match perfectly the positions of all observed peaks in the spectrum. In particular, Figure 12a clarifies why, for decreasing *v*_SL_, new excited phonon modes always arise near, but not quite at, the BZ boundary.

**Figure 13:**
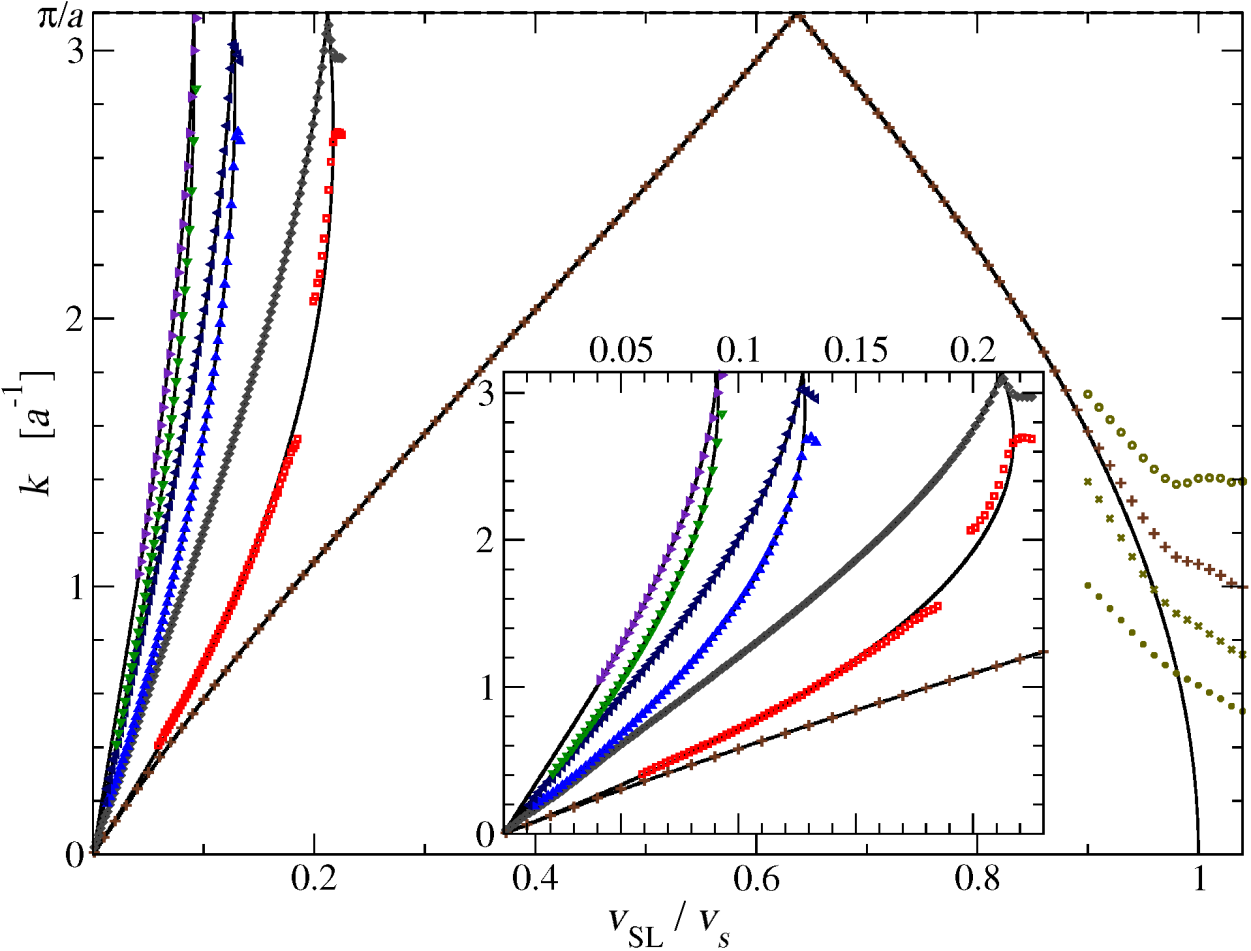
The positions of the observed peaks of 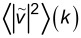 (symbols), compared with the values of *k* of the phonons whose phase velocity is equal to *v*_SL_ (black solid lines), solutions of Equation 14. Inset: a blow up of the interval 0 ≤ *v*_SL_ ≤ 0.23 *v_s_*.

Figure 13 compares the positions of the peaks of 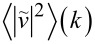 obtained from the analysis of MD trajectories as in Figure 10, with the wave vectors of the phonons whose phase velocity equals *v*_SL_, obtained by solving Equation 14. It is apparent that for *v*_SL_ 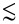 0.9 *v_s_* the two quantities match almost perfectly. We conclude that Equation 14 provides a reliable prediction of the wave vectors of the excited phonons for a given *v*_SL_. Above the speed of sound *v_s_*, Equation 14 has no solution and indeed the spectrum displays no sharp peak, see Figure 9a. Correspondingly the “nonresonant” friction is very small, see the right side of Figure 6.

In particular Equation 14 allows us to predict the number and wave vectors of the excited phonon modes when the slider is moving at a given velocity. The number *N*_ph_ of excited phonon modes at a given *v*_SL_ is null for any *v*_SL_ *> v_s_*, it turns to unity for 0.217 *v_s_* *< v*_SL_ *< v_s_*, and then for smaller and smaller *v*_SL_ *N*_ph_ increases by two every time the dashed line of Figure 12a crosses one more sine hump. As a result, in the limit of small *v*_SL_, *N*_ph_ is approximately

[16]
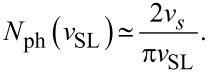


We have then a number of excited phonons which is inversely proportional to *v*_SL_ for small *v*_SL_. The comparison of Figure 11 suggests that an increasing number of excited phonon modes leads to an increasing kinetic friction *F_d_*, see Figure 11. If a comparable power was dissipated in each of the excited phonon modes, then Equation 16 would be compatible with the power-law dependence *F_d_*(*v*_SL_) 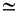 *A*/*v*_SL_ that we observe in the low-speed regime in Figure 6. However there is no evidence that the same power is dissipated in each one of the phonon modes. In fact, Figure 10b,d shows that different modes draw different spectral weights. A systematic study of the intensities of the phonon excitations would probably be useful in understanding the *F_d_*(*v*_SL_) 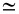 *A*/*v*_SL_ behavior in detail.

## Discussion and Conclusion

We introduce a new model, or rather a model class, that allows one to investigate friction at the very point where it is generated: the contact point. In the present paper we examine the main predictions of this model in its most basic incarnation: a point-like slider kept at a fixed distance to a 1D harmonic chain of masses and springs. The main result is that kinetic friction should depend on speed as a generally decreasing function, with several nontrivial features deviating from monotonicity: friction peaks arise every time a new “resonance condition” is fulfilled, namely that the phase velocity of some substrate phonon mode matches the slider speed. Recently published research also identified relations between dissipation peaks and the properties of a dispersion relation in a different model [63].

The present model can also be investigated in a spring-pulling scheme analogous to the Prandtl–Tomlinson model, to simulate the finite stiffness of an AFM cantilever. In that scheme a stick-slip to smooth-sliding transition can also be investigated, especially at low speed (see Appendix “The static friction force”), allowing one to study in detail the nonlinear phenomena and mechanisms of phonon excitations that arise at slip times. The stick-slip regime and the associated nonlinear phenomena will of course be more significant when the model is studied in a realistic regime of intermediate to strong coupling ε ≈ *Ka*^2^. We defer this investigation to future work, which should provide a microscopic basis and the limits of applicability for the Prandtl–Tomlinson model. This simple 1D model, studied in the spirit of protocol B (fixed slider speed) for weak slider-chain coupling ε 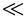 *Ka*^2^, should be suitable to address even by means of analytic perturbative many-body methods. We plan to pursue this investigation in the future.

The FK model and the present model both describe dissipation in terms of the excitations of the phonon modes of a linear harmonic chain. Therefore, the two models agree that no resonant energy transfer can occur for very large speed, and friction drops to 0, as at the right side of Figure 6. However, the frictional features demonstrated in the present work differ significantly from those found for the superlubric weak-corrugation regime of the FK model [25,43]. Specifically, in the FK model the condition for dissipation peaks, where center-mass translational energy is converted rapidly into internal phononic degrees of freedom, requires that the modulation dictated by the chain-potential lattice incommensurability resonates with the washboard frequency at the center-mass sliding velocity, due to the sinusoidal potential acting to perturb the entire harmonic chain at a single wave vector. That conditition reflects the extended nature of the contact, and may be suitable to describe friction as generated at crystalline incommensurate solid-solid interfaces. Instead, in the present model dissipation occurs simultaneously through whatever phonon has a phase velocity matching the slider speed, and resonant peaks arise as more and more phonons become available at slower speeds. In the current model, more suitable to describe the dissipation of an AFM tip, there is no equivalent to the relative spatial periodicity, with the effect that phonons at all wave vectors can virtually be excited simultaneously. As a result, the present model exhibits dissipation peaks of the same physical nature (i.e., phononic excitations) but in detail quite different from those of the FK model. Relatedly and importantly, the obtained low-speed behavior, with friction increasing and approaching the static limit when the sliding velocity decreases to 0 is opposite to that obtained for the single-wave vector FK model.

More refined incarnations of this model are interesting targets of future study. We mention just a few obvious generalizations: (i) remove the constraint of a fixed slider-chain distance, thus allowing for longitudinal-to-transverse energy transfer; (ii) replace the point slider with a more realistically structured slider consisting of several atoms, e.g., placed periodically, thus making contact with the FK model, or rather in a disordered structure; (iii) replace the 1D chain by a 2D [64], or a more realistic 3D harmonic crystal; (iv) adopt, rather than a harmonic crystal, a realistic force field modeling a specific substrate, with the target of predicting the frictional dynamic features of a specific interface as was done, e.g., in [49,65]; (v) energy dissipation implemented through suitably remote boundary-atom thermostats [30–31,33] rather than via an unphysical damping throughout the crystal.

In the present work we do our best to keep the chain as close as possible to its *T* = 0 ground state. The study of this or any of the generalizations of this model at finite temperature will also provide a playground to investigate all sorts of thermolubric effects [25,45–46,57–62]. It is presently unclear which of the predictions of the simple 1D model studied here will hold in the extended versions of the model, and at finite temperature. It will be particularly interesting to find out how dissipation changes when energy is transferred to a physically realistic density of phonon modes of 3D crystals, rather than 1D.

## Appendix: the Static Friction Force

**Figure 14:**
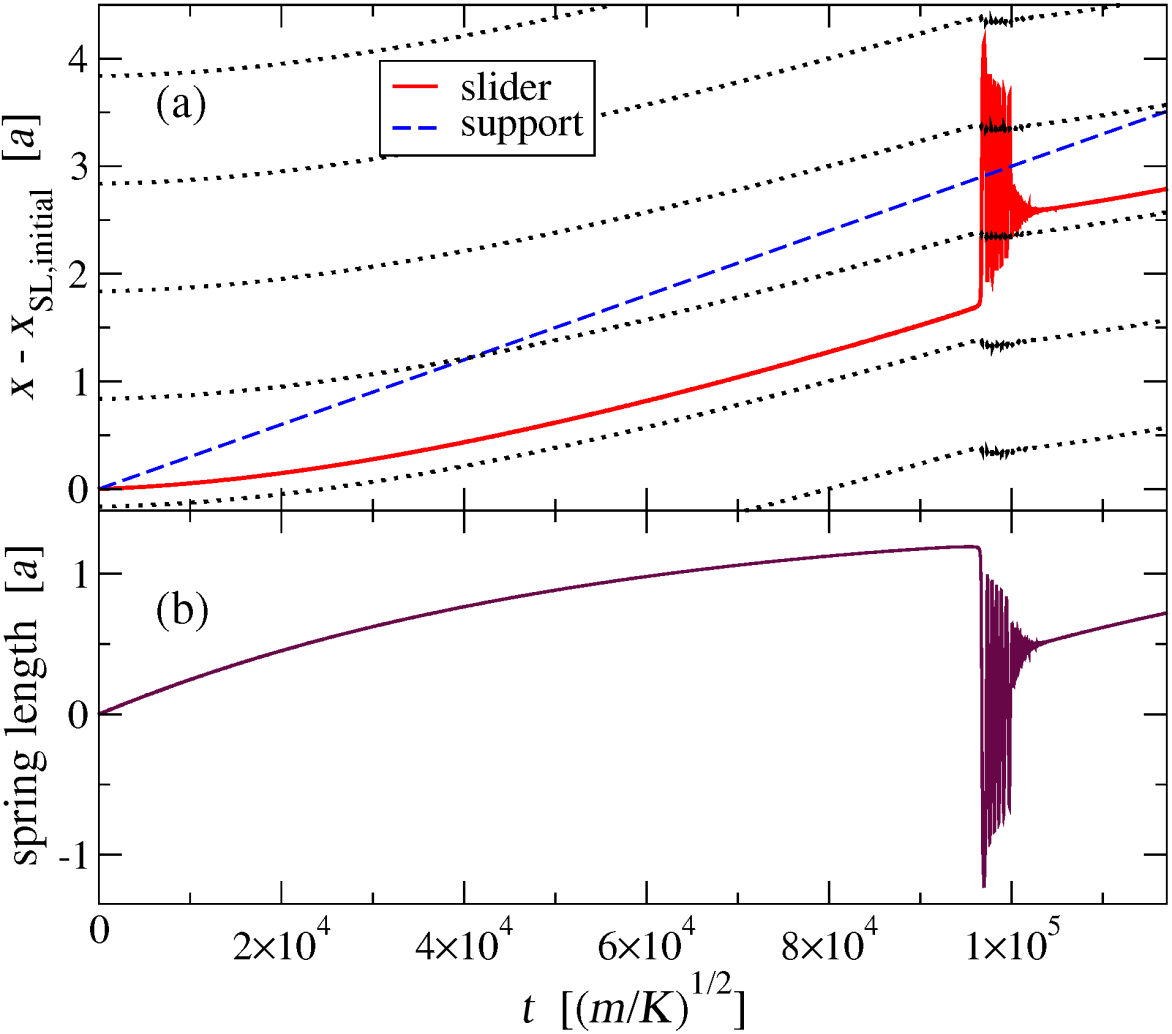
(a): The motion of the slider (solid line) as it is pulled by a spring having elastic constant *K*_pull_ = 10^−3^ *K* connecting it to a support (dashed line) advancing at a constant speed *v*_pull_ = 3 × 10^−5^ *v_s_*; the dotted lines mark the time evolution of the positions *x_j_* of successive atoms in the chain. (b): The pulling spring length as a function of time: the spring elongates until the slider suddenly slips forward, oscillates around the next stick position, and dissipates the energy accumulated in the pulling spring by emitting phonons. The process then repeats itself.

The solid curve of Figure 2 shows two kinds of nonequivalent equilibrium positions: immediately at the left and at the right of a chain atom. Obviously, the most robust of them against rightward sliding is the one of them sitting at the right of an atom, thus we probe the static friction there. For this purpose, we place the slider at that minimum, and run a simulation where we pull the slider through a spring of elastic constant *K*_pull_ extending from the slider to a support which moves at a constant velocity *v*_pull_ to the right. Figure 14a reports the positions of the support, the slider, and a few chain atoms as a function of time. At the beginning, the spring elongates (Figure 14b) while the slider remains stuck near the minimum. Meanwhile, as a result of the force between the slider and the atoms, the whole chain accelerates slowly to the right. If *v*_pull_ is large enough, there comes a time at which the pulling spring elongates enough for the pulling force to exceed the static friction threshold *F*_static_, and the slider suddenly abandons its initial position near a certain atoms, oscillates around the support dissipating the elastic energy accumulated in the pulling spring, and eventually reaches a quasi-stationary “stick” configuration at a new local equilibrium position near the next atom at the right of the starting one. Then the stick-slip process repeats itself. Conversely, if *v*_pull_ is less than a certain critical value *v*_pull c_, the whole chain speed advances at a speed asymptotically close to *v*_pull_: the support, the slider, and the chain slide together at the same velocity and the spring never elongates enough for the elastic force to exceed *F*_static_.

**Figure 15:**
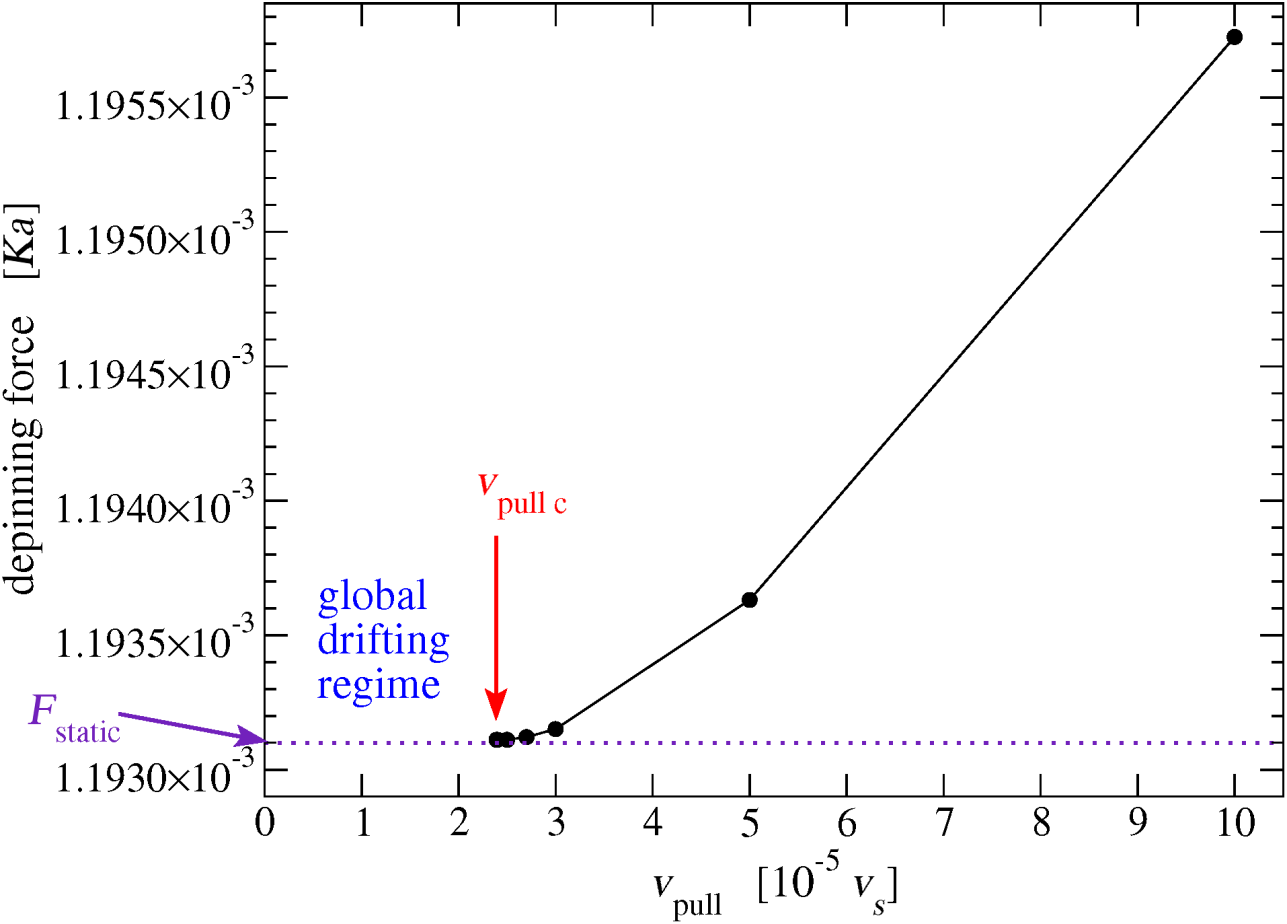
For smaller and smaller support velocity *v*_pull_, the maximum elastic force experienced by the slider just before it overtakes one atom of the chain provides a better and better estimation of the static friction *F*_static_. By moving closer and closer to the minimum *v*_pull c_, we obtain a quantitative estimate of the actual *F*_static_, see Equation 9. In these simulations, the pulling elastic constant *K*_pull_ = 10^−3^ *K*.

In simulations with *v*_pull_ greater than the (hitherto undetermined) critical speed *v*_pull c_, the pulling spring length reaches a maximum value before the slider abandons its equilibrium position. By multiplying this maximum elongation by *K*_pull_, we obtain the maximum pulling force experienced by the slider, which systematically overestimates *F*_static_. We repeat this calculation for decreasing values of *v*_pull_, and record the resulting estimations of *F*_static_ in Figure 15. Our resulting best estimation for the static friction is

[9]
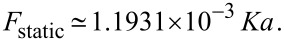


The transition from the sliding regime to the low-speed global drift mode, Figure 15, provides the following estimation for *v*_pull c_ = (2.385 ± 0.005) × 10^−5^ *v_s_*. An even better estimation of *v*_pull c_ can be obtained from *F*_static_ by a simple relation with the damping terms of Equation 6 hindering the chain advancement. In low-speed *v*_pull_ *< v*_pull c_ regime the chain, the slider and the pulling support all drift rightward together at the same speed *v*_pull_. This constant-speed advancement requires a null total force acting on the chain. Accordingly, the total force *F*_SL−chain_ that the slider exerts on the chain must be equal in strength but opposite in direction to the total damping force of Equation 6, which is *N*γ*v*_pull_:

[17]
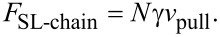


The maximum speed *v*_pull_ for which this situation occurs is *v*_pull c_, which corresponds to the maximum possible value of *F*_SL−chain_, namely *F*_static_. Then, at the depinning transition Equation 17 requires

[18]
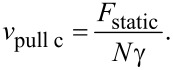


In the conditions of our simulations we obtain

[19]
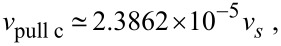


perfectly compatible with the previous estimation. While, according to Equation 18, *v*_pull c_ is strongly dependent on simulation details such as *N* and γ, *F*_static_ is not, and is therefore a physically significant quantity. The obtained *F*_static_ value is of course dependent on the precise slider-substrate interaction, which in the present model is tuned by the σ and *d* lengths, and by the LJ coupling energy ε. For small ε 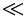 *Ka*^2^, *F*_static_ is essentially proportional to ε.
